# Immuno-antioxidative reno-modulatory effectiveness of *Echinacea purpurea* extract against bifenthrin-induced renal poisoning

**DOI:** 10.1038/s41598-024-56494-4

**Published:** 2024-03-11

**Authors:** Khaled G. Abdel-Wahhab, Ghada M. Elqattan, Doaa G. EL-Sahra, Laila K. Hassan, Rehab S. Sayed, Fathia A. Mannaa

**Affiliations:** 1 Medical Physiology Department, National Research Centre, Giza, 12622 Egypt; 2 Modern University for Technology and Information, Cairo, Egypt; 3 Dairy Department, National Research Centre, Giza, 12622 Egypt; 4 Regional Center for Food and Feed, Agriculture Research Centre, Giza, Egypt

**Keywords:** Biochemistry, Chemical biology, Physiology, Medical research, Nephrology

## Abstract

This study was conducted to evaluate the ameliorative, anti-inflammatory, antioxidant, and chemical detoxifying activities of *Echinacea purpurea* ethanolic extract (EEE) against bifenthrin-induced renal injury. Adult male albino rats (160–200 g) were divided into four groups (10 rats each) and orally treated for 30 days as follows: (1) normal control; (2) healthy animals were treated with EEE (465 mg/kg/day) dissolved in water; (3) healthy animals were given bifenthrin (7 mg/kg/day) dissolved in olive oil; (4) animals were orally administered with EEE 1-h prior bifenthrin intoxication. The obtained results revealed that administration of the animals with bifenthrin caused significant elevations of serum values of urea, creatinine, ALAT and ASAT, as well as renal inflammatory (IL-1β, TNF-α & IFN-γ), apoptotic (Caspase-3) and oxidative stress (MDA and NO) markers coupled with a marked drop in the values of renal antioxidant markers (GSH, GPx, and SOD) in compare to those of normal control. Administration of EEE prior to bifenthrin resulted in a considerable amelioration of the mentioned deteriorated parameters near to that of control; moreover, the extract markedly improved the histological architecture of the kidney. In conclusion, *Echinacea purpurea* ethanolic extract has promising ameliorative, antioxidant, anti-inflammatory, renoprotective, and detoxifying efficiencies against bifenthrin-induced renal injury.

## Introduction

Synthetic pyrethroids (SPs) are the most frequently utilized type of insecticides. They represent over 25% of the worldwide pesticide industry. Because of the toxic effects of these insecticides on human and animal, the Environmental Protection Agency restricted most residential applications of them; however, they are still utilized up to date^1^.

Bifenthrin, a third generation type1 of SP, has received a widespread attention due to its superior insecticidal action and stability when compared to previous SPs^2^. It is utilized for controlling flies, mosquitoes, cockroaches, and termites, by interference with ATPase enzymes and voltage-gated ion channels, resulting in hyperexcitability, tremors, convulsions, and death. Furthermore, it has been reported that bifenthrin-exposure causes neurotoxic effects in mammals’ brains^3^, as well as apoptotic and inflammatory responses in murine brains^4,5^. Also, it was reported that bifenthrin is more hazardous than other SPs as it stays longer in the soil, quickly attaches to organic materials, and flows into aquatic areas, causing ecological dangers and harmful impacts on aquatic life^6^.

Bifenthrin has been proven to be highly poisonous, tumorigenic, immunotoxic, and hepatotoxic in many species of mammals via oxidative stress pathways, and/or hormonal-disrupting influence^7^. Moreover, its chronic poisoning in animals results in tremor, hypersensitivity to stimuli, and aggressive sparring**.** In addition, there is an aggressive danger due to the long-term exposure to its residues in food products for humans^8^. Bifenthrin induces oxidative stress in animal models resulting in both increased oxidant markers and decreased antioxidant activities^9^.

Medicinal plants are still considered important and promising sources of drugs to treat various diseases. Traditional medicine is the cornerstone that boosts scientific research to explore new therapeutic approaches. Pathophysiological-modulating properties of plants are being studied extensively to achieve their desired impacts on disease prevention; as a result, herbal therapies have been used over decades for their efficacy, safety, minimal adverse effects, and cultural acceptance^10^.

*Echinacea* genus plants, popularly referred to as coneflower, are prominent medicinal plants of the *Asteraceae* family that originated in Russia, Canada, Australia, and United States^11^. They were reported to have inhibitory, antiproliferative, antibacterial, and antioxidant activities. *Echinacea purpurea* (*E. purpurea*) is one of the most often used plant species in medicine. In many circumstances, *E. purpurea* L. is used as food-supplementation for the treatment of a variety of disorders, including respiratory tract infections, immune system strengthening, wound healing, migraine headaches discomfort, and anxiety relieve. It is available in teas, creams, lotions, tablets, capsules, and other forms^12^. *E. purpurea* has attracted international attention in the past few years^13^. The plant extracts provide antifungal, antiviral, antibacterial, and antioxidative activities; moreover, they are utilized for treating the common cold, in addition to urinary illnesses^14^. Cichoric acid, echinacoside, chlorogenic acid, caffeic acid, cynarine, sitosterol, polyacetylene, alkylamides, alkaloids, glycosides, and carbohydrates (active ingredients of *E. purpurea*) are all strong antioxidants that can successfully eliminate free radicals as well as decrease the nitric oxide free radical generation^15^. Also, the plant includes prevalent ingredients including lipoproteins, polysaccharides, caffeic acid derivatives, and alkylamides; however, these active phytochemicals differ based on how old the plant is, section of the plant, growing circumstances, geographical region, and extraction process. Polysaccharides are often found at the highest levels in aqueous or freshly squeezed juice extracts, whereas alkylamides have a greater tendency to be key ingredients in ethanoic extracts^16^.

Many studies have demonstrated that bifenthrin exposure is toxic to numerous organs of the body, such as brain, testes, kidneys, lungs, and liver^17^; consequently, the key objective of this study was to evaluate the ameliorative potential, and to investigate the anti-inflammatory, antioxidant, and detoxifying activities of *E. purpurea* ethanolic extract against bifenthrin-induced renal injury.

## Materials and methods

All the used chemicals (pure and of analytical grade) were obtained from the stores of the National Research Centre, Egypt. Bifenthrin (purity 98%) was purchased from Sigma, MO, USA. Flowers of *E. purpurea* were obtained from IMTENAN Company (Production and sale of nutritional and healthy products, Giza, Egypt); the company collected and dried the flowers in accordance with relevant institutional, national, and international guidelines and legislation. The plant was examined by botanical specialists (Faculty of pharmacy, Ain-Shams university, Cairo, Egypt) and was found carrying the taxonomic serial number 3728.

### Extraction of the herb

The plant flowers were air-dried andn grinded; then two hundred grams from the powdered material were soaked in one liter of 70% ethanol for seven days. After filtration through Whatman 1 filter paper, the filtrate was evaporated using rotary evaporator. The remaining moisture residue was lyophilized by freeze drier^18^. Finally, the obtained dry extract (EEE) was stored at – 80 °C until further use.

### HPLC analysis of phenolic ingredient

HPLC analysis was carried out using an Agilent 1260 series. The separation was carried out using Eclipse C18 column (4.6 mm × 250 mm i.d., 5 μm). The mobile phase consisted of water (A) and 0.05% trifluoroacetic acid in acetonitrile (B) at a flow rate 0.9 ml/min. The mobile phase was programmed consecutively in a linear gradient as follows: 0 min (82% A); 0–5 min (80% A); 5–8 min (60% A); 8–12 min (60% A); 12–15 min (82% A); 15–16 min (82% A) and 16–20 (82%A). The multi-wavelength detector was monitored at 280 nm. The injection volume was 5 μl for each of the sample solutions. The column temperature was maintained at 40 °C.

### Experimental design

Forty adult male albino rats (160–200 g) were provided from the animal house colony, National Research Centre. They placed in suitable plastic cages and fed with standard rodent pellets [20.3% protein (20% casein and 0.3% DL-Methionine), 5% fat (corn oil), 5% fibers, 3.7% salt mixture and 1% vitamin mixture, obtained from Meladco Company, El-Obour City, Cairo, Egypt] and water ad libitum one week for acclimatization before starting the experiment. All animals received human care in compliance with the standard institution’s criteria; the proposal was approved and certified by the ethics committee of the use of experimental animals, Faculty of Science, Al-Azhar University with a reference number (#AZHAR 9/2023, Feb 2023).

After being acclimatized, the animals were divided into 4 groups (10 rats each group) as follows: group (1) healthy rats ingested with 2 ml of water and, served as normal control; group (2) healthy rats orally given EEE dissolved in water (465 mg/kg/day) as described by Mao et al.^19^ [fixed concentration of EEE in water was prepared, then each rat was administrated with a volume containing the weight-equivalent dose of EEE]; group (3) normal rats orally intoxicated with bifenthrin (7 mg/kg/day) dissolved in olive oil as reported by Syed et al.^5^ [Similarly, fixed concentration of bifenthrin in olive oil was prepared, then each rat was treated with a volume containing the weight-equivalent dose of bifenthrin]; finally group (4) rats administrated with EEE (same dose of group 2) one-hour prior to bifenthrin (same dose in group 3) intoxication.

### Blood and tissue sampling

At the end of the experimental duration, the rats were fasted overnight, and anesthetized, then blood samples were collected, left to clot, and cool-centrifugated at 3000 rpm for 15 min; then, the clear sera were separated and stored at − 80 °C until determination of liver function (serum ALAT and ASAT activities) and kidney function (serum urea and creatinine levels). After blood collection, the animals were sacrificed soon by sudden decapitation; then both kidneys (of each rat) were removed; the left kidney was washed in saline, dried with filter paper, rolled in a piece of plastic sheet, and stored at – 80 °C for determination of inflammatory and oxidative stress markers, and the percentage of DNA fragmentation. The right one was immersed in 10% formalin-saline (v/v) buffer for histopathological examination.

### Serum biochemical measurements

Serum alanine aminotransferase (ALAT), aspartate aminotransferase (ASAT), urea and creatinine were estimated using reagent kits purchased from Bio Vision, Inc, USA.

### Preparation of kidney homogenate

The kidney of each animal was ultrasonically homogenized (SONICS homogenizer, France) in phosphate buffer (0.1 M, pH 7.4) at ratio of 1:10 (w/v), then cool centrifuged (Hettich centrifuge, NEWTOWN CT, USA) at 5000 rpm for 10 min. The clear supernatant separated, divided into aliquots, and stored at − 80 °C until used for determinations of renal oxidative stress markers and cytokines.

### Determination of renal inflammatory and apoptotic markers

The levels of renal interleukin-1beta (IL-1β), tumor necrosis factor alpha (TNF-α) and Caspase-3 were estimated using rats’ reagent ELISA kits obtained from Sino Gene Clon Biotech Co., Hang Zhou, China, while the assessment of interferon-γ (IFN-γ) level was carried out using rats’ reagent ELISA kits purchased from Ray Biotech Co., Norcross, Georgia, USA.

### Estimation of renal oxidative stress markers

Renal lipid peroxidation (Malondialdehyde, MDA), nitric oxide (NO) and reduced glutathione (GSH) levels as well as enzymatic activity of glutathione peroxidase (GPx), and superoxide dismutase (SOD) in renal tissue were determined spectrophotometrically using reagent kits obtained from Bio diagnostic Co., Giza, Egypt.

### Determination of DNA fragmentation

The degree of the DNA fragmentation was determined using the quantitative method used for grading the DNA damage as described by Perandones et al.^20^. The degree of DNA fragmentation was determined by separating the cleaved DNA from the intact chromatin by centrifugation and measuring the amount of DNA present in the supernatant and pellet using the diphenylamine assay. The percentage of the fragmented DNA was calculated as follow:

$$\mathrm{DNA\,fragmentation \% }= \frac{\mathrm{A\,supernatant }}{\mathrm{A\,supernatant}+\mathrm{A\,pellet }} \times 100$$

### Histopathological examination

Kidney samples were hydrated in ascending grades of ethanol, cleared in xylene, and embedded in paraffin. Sections of 5 μm thick were cut and stained with hematoxylin and eosin for histological examination under light microscopy^21^.

### Histopathological scoring

The histopathological scoring was conducted in a blinded fashion, wherein the assessment of pathological alterations, including tubular necrosis, cast formation, brush border loss, and tubular dilatation, was carried out in 10 randomly selected, non-overlapping fields at a magnification of 400× . This grading system ranged from 0 (absent) to 5 (present in ≥ 76%), as outlined in the seminal work by Chang et al.^22^.

### Morphometric analysis

An image analysis system consisting of a digital camera and a light microscope was used for histomorphometry. The evaluation focused on studying glomerular injury in H&E-stained kidney sections (5 μm thick). Digital images of thirty glomeruli per animal were captured using a light microscope. Then, a computerized morphometric analysis system (ImageJ; National Institutes of Health) was employed to trace and calculate the Bowman's capsule area (BCA), glomerular tuft area (GTA), and Bowman’s space area (BSA) from the digitized images.

### Statistical analysis

Multiple comparisons between means were carried out using one-way ANOVA followed by Duncan post hock test at *p* ≥ 0.05 using statistical analysis system (SAS) program software; copyright (c) 1998 by SAS Institute Inc., Cary, NC, USA.

### Ethical approval

All methods and the experimental animals were used in accordance with the standard criteria of the ethics committee, Faculty of Science, Al-Azhar University, Egypt that approved the used methods, experimental animals, and protocols with a reference number (#AZHAR 9/2023, Jan. 2023). The authors confirm that the study was carried out comply with the ARRIVE guidelines.

## Results

### In vitro study

**Figure 1:**
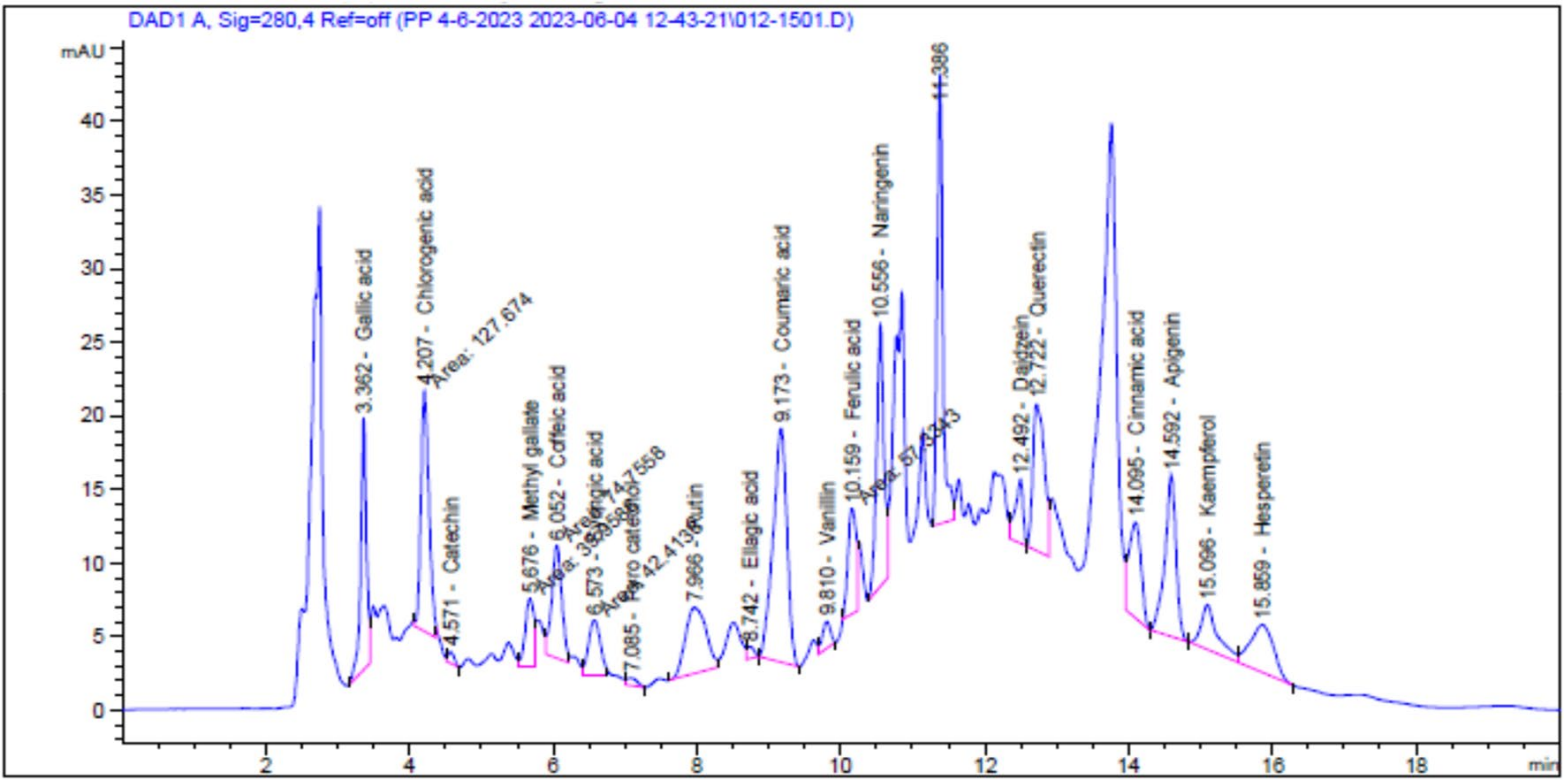
HPLC imprint profile of EEE. 19 components were identified, other peaks were not identified.

The in vitro analyses of the EEE revealed that the extract contains significant amount of total phenolic compounds and has a strong antioxidant capacity because of its strong capacity to remove DPPH radicals, which validates the consequent reducing power, which indicated a concentration-dependent relationship. In addition, the phytochemical analysis of the EEE by HPLC revealed the identification of 19 phenolic and flavonoid compounds. Among these, chlorogenic acid, naringenin, gallic acid coumaric acid, and caffeic acid were the major phenolic components while querectin, rutin, and apigenin were the major flavonoids compound present in the extract (Table 1 and Fig. 1).

**Table 1:** HPLC analysis of EEE.

	Area	Concentration (µg/ml = 20 mg/ml)	Concentration (µg/g)
*Phenolic acids*			
Gallic acid	94.15	8.13	406.51
Chlorogenic acid	127.67	17.48	874.19
Caffeic acid	74.76	5.75	287.44
Syringic acid	42.41	2.88	143.80
Ellagic acid	5.46	1.01	50.63
Coumaric acid	214.12	6.76	337.75
Ferulic acid	57.33	3.92	195.86
Cinnamic acid	76.35	1.41	70.33
*Flavonoids*			
Catechin	4.91	1.21	60.74
Methyl gallate	39.96	2.18	109.05
Pyro catechol	5.23	0.75	37.62
Rutin	89.65	10.41	520.50
Vanillin	14.68	0.64	32.13
Naringenin	124.86	15.06	752.83
Daidzein	33.05	2.04	102.08
Querectin	111.91	15.42	770.75
Apigenin	118.19	9.00	449.84
Kaempferol	48.40	3.75	187.65
Hesperetin	72.99	4.25	212.75

### In vivo study

Our data illustrated that the levels of ALAT, ASAT, urea, and creatinine in the healthy rats were not affected by the administration with EEE; this demonstrates the biological safe effect of EEE. In contrast, administration of the animals with bifenthrin resulted in a significant increase in the values of these measurements confirming the toxic effect of bifenthrin. Favorably, administration of animals with EEE one-hour prior to bifenthrin intoxication led to a marked downregulation of ALAT, ASAT, urea, and creatinine values near to the corresponding values of the normal control. This proves the protecting effect of EEE against bifenthrin exposure (Table 2).

**Table 2:** Effect of *E. purpurea* ethanolic extract (EEE) on liver functions (ASAT and ALAT) and kidney functions (urea and creatinine) in rats treated with bifenthrin.

	ALAT (U/ml)	ASAT (U/ml)	Urea (mg/dl)	Creatinine (mg/dl)
Control	36.22 ± 1.81	33.55 ± 1.1	29.56 ± 1.95	0.783 ± 0.03
EEE	34.65 ± 1.75^ns^	32.15 ± 1.33^ns^	28.25 ± 1.27^ns^	0.724 ± 0.028^ns^
Bifenthrin	117.3 ± 3.26*	95.64 ± 2.88*	83.5 ± 3.65*	2.34 ± 0.054*
EEE + Bifenthrin	55.32 ± 2.11^**#**^	62.38 ± 2.10^**#**^	51.65 ± 2.71^**#**^	1.35 ± 0.041^**#**^

Data are presented as mean ± standard error. Within the same column, means with superscript symbol (*) is significantly and (ns) is non-significantly different from of control; while those with superscript symbol (**#**) is significantly different from bifenthrin intoxicated group at *p* ≤ 0.05; EEE (*E. purpurea* ethanolic extract).

In the current study, administration of the healthy rats with EEE did not disturb the renal inflammatory (IL-1β, TNF-α, &IFN-γ) and apoptotic (Caspase-3) markers; this confirms the safe effect of EEE on the renal; while intoxication of animals with bifenthrin caused significant increases in the levels of these markers when both groups were compared with the corresponding values of control. These marked increments were significantly returned, close to the corresponding levels of normal control, in the fourth group in which the animals were treated with EEE prior to bifenthrin intoxication; this result reflects the anti-inflammatory and anti-apoptotic effects of EEE in the intoxicated rats (Table 3).

**Table 3:** Effect of *E. purpurea* ethanolic extract (EEE) on IL-1β, TNF-α, IFN-γ, and caspase-3 in animals treated with bifenthrin.

	IL-1β (ng/g tissue)	TNF-α (µg/g tissue)	IFN-γ (pg/g tissue)	Caspase-3 (U/g tissue)
Control	84.1 ± 6.4	112.2 ± 3.8	978 ± 8.8	503 ± 1.21
EEE	82.3 ± 7.1^ns^	109.4 ± 4.3^ns^	990 ± 7.9^ns^	522 ± 1.07^ns^
Bifenthrin	221.5 ± 9.8*	266.1 ± 8.2*	2175 ± 11.5*	1758 ± 12.3*
EEE + Bifenthrin	113.8 ± 7.7^#^	145.6 ± 6.9^#^	1354 ± 9.9^#^	844 ± 7.6^#^

Data are presented as mean ± standard error. Within the same column, means with superscript symbol (*) is significantly and (ns) is non-significantly different from of control; while those with superscript symbol (**#**) is significantly different from bifenthrin intoxicated group at *p* ≤ 0.05; EEE (*E. purpurea* ethanolic extract).

Regarding renal oxidative stress status, the present study showed that administration of rats with EEE never disturb the oxidative stress status of the renal tissue, while a marked elevation in the levels of renal oxidative (MDA and NO) markers matched with a sharp reduction in the values of renal antioxidant (GSH, GPx & SOD) markers was noticed in the bifenthrin-intoxicated group in compared to normal control. Fortunately, treatment of rats with EEE before bifenthrin ingestion resulted in an obvious upregulation in the antioxidant indicators (GSH, SOD & GPx) coupled with a notable downregulation in MDA and NO (Table 4).

**Table 4:** Effect of *E. purpurea* ethanolic extract (EEE) on renal MDA, NO, GSH, GPx, and SOD values in animals treated with bifenthrin.

	MDA (nmol/g tissue)	NO (µmo1/g tissue)	GSH (mmo1/g tissue)	GPx (U/g tissue)	SOD (U/g tissue)
Control	146.5 ± 4.3	53.7 ± 2.1	4.82 ± 0.86	4873 ± 32.5	8254 ± 66.5
EEE	140.3 ± 3.2^ns^	49.8 ± 2.3^ns^	5.01 ± 1.11^ns^	4921 ± 41.2^ns^	8377 ± 73.2^ns^
Bifenthrin	387 ± 13.5*	216 ± 9.3*	1.97 ± 0.35*	2049 ± 23.7*	3746 ± 44.5*
EEE + Bifenthrin	194.6 ± 8.7^#^	74.5 ± 4.7^#^	3.42 ± 0.77^#^	3894 ± 29.8^#^	6668 ± 59.6^#^

Data are presented as mean ± standard error. Within the same column, means with superscript symbol (*) is significantly and (ns) is non-significantly different from of control; while those with superscript symbol (**#**) is significantly different from bifenthrin intoxicated group at *p* ≤ 0.05; EEE (*E. purpurea* ethanolic extract).

**Figure 2:**
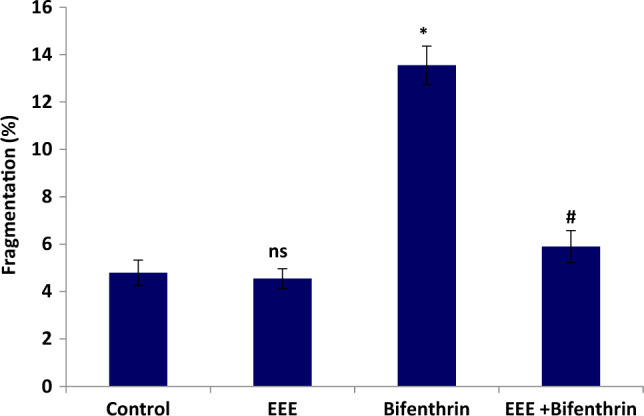
Effect of EEE on renal DNA fragmentation. Data are presented as mean ± standard error. The symbol (*) is significantly and (ns) is non significantly different from that of the control, while those with the symbol (#) is significantly different from bifenthrin intoxicated group at *p* ≤ 0.05; EEE is *E. purpurea* ethanolic extract.

The effect on renal DNA fragmentation percentage is shown in Fig. 2. The current study illustrated that ingestion of EEE did not perform any damaging effect on DNA, while bifenthrin intoxication caused a significant increase in the DNA fragmentation percentage in compare to the control group. In a promising manner, administration of rats with EEE one hour prior to bifenthrin successfully reduced DNA fragmentation value in compare to bifenthrin intoxicated group.

### Histopathological and morphometric evaluations

**Figure 3:**
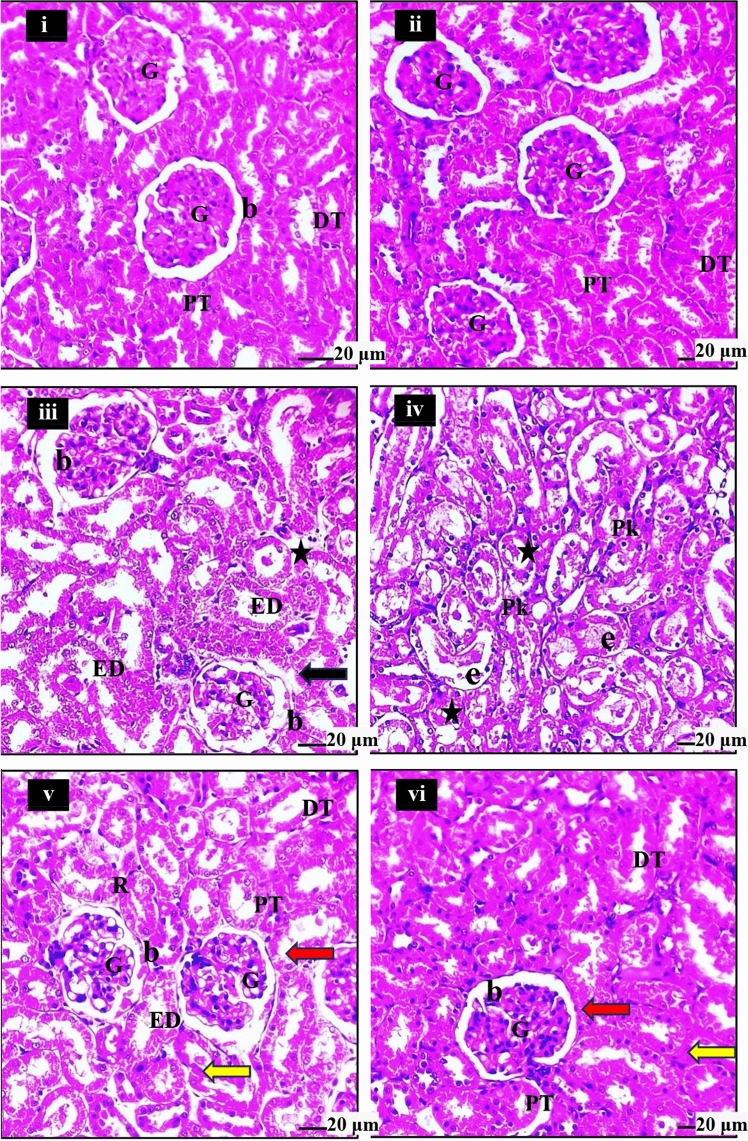
Photomicrographs of kidneys of normal and treated animals’ groups. (i) is the image of the normal control group (group 1); it demonstrates normal renal structures. (ii) is the image of EEE administrated group (group 2); the image shows almost normal kidney structures. In contrast, (iii) and (iv) images are different sections of bifenthrin-intoxicated group (group 3); the image (iii) shows severe renal damage (black arrow) and necrosis (star), while the image (iv) illustrates necrosis (star); Finally, (v) and (vi) are images of the therapeutic group (treated with EEE post-bifenthrin intoxication) (group 4) the images demonstrate clear improvements in the microanatomical structure of glomeruli (red arrow), and in renal capsules (yellow arrow), displaying normal glomeruli and Bowmans spaces. The abbreviations used in the figure include G for glomeruli, PT for proximal tubule, DT for distal tubule, b for Bowman’s space, e for eosinophilic cast, ED for epithelial degeneration, and PK for pyknosis. (Hematoxylin and Eosin, Magnification power =  × 200, Scale bare = 20 μm).

The assessment of kidney sections through histopathological examination was conducted in this study. The kidney sections from the normal control (group 1) displayed a normal architecture in the outer cortex and inner medulla, i.e. showed typical and healthy structures (Fig. 3i); while the group treated with EEE (group 2) displayed a histological framework almost similar to the control group; morphometric evaluations showed no significant differences in the glomerular, tuft, and Bowmans space areas between the normal and EEE-treated groups (Fig. 3ii). In contrast, the group intoxicated with bifenthrin (group 3) showed severe renal damage and necrosis, with significant histopathological changes, including degeneration, dilatation of Bowman’s capsule, glomerular atrophy, and disintegration of the tubular epithelium, pyknotic nuclei, and proteinaceous casts in the renal tubules. Morphometric evaluations revealed an increase in the glomerular and Bowmans space areas (Fig. 3 iii and iv). Post-treatment of the bifenthrin-intoxicated rats with EEE (group 4) resulted in a significant improvement in the microanatomical structure of the glomeruli, with the elimination of eosinophilic cast formations and concurrent regeneration of glomeruli in the basal cortical and juxtamedullary regions. Furthermore, a remarkable improvement in the renal capsules, displaying near-normal appearances as well as the renal tubules showing marked improvements (Fig. 3 v and vi).

**Figure 4:**
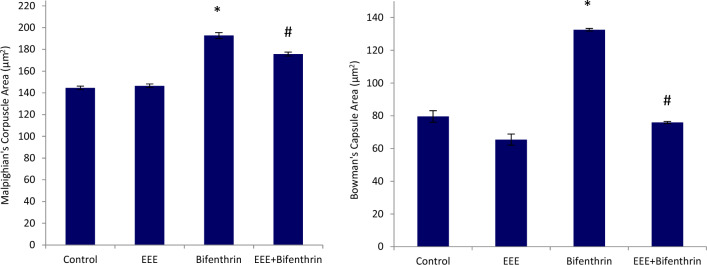
Depiction of the examination of kidney tissue microanatomy in rats treated with various substances. The morphometric analysis showed significant increases in these areas compared to the healthy control group. However, group 4 demonstrated improvements in the glomerular and Bowman’s space areas compared to the bifenthrin-treated group, indicating possible therapeutic effects. The symbol * is significantly different from that of the control, while those with the symbol # is significantly different from bifenthrin intoxicated group at *p* ≤ 0.05; EEE (*E. purpurea* ethanolic extract).

The morphometric analysis showed that the normal group (group 1) displayed healthy structures, such as typical tubules, glomerular capillaries, Malpighian corpuscles, and Bowman’s capsule. Kidney sections from rats treated with the EEE (group 2) displayed almost normal histological features, akin to those of the control rats. In contrast, group 3, treated with bifenthrin, exhibited severe renal damage and necrosis, as evidenced by marked changes in the glomerular and Bowmans space areas. group 4 showed a significant improvement in the microanatomical structure of the glomeruli, and a remarkable improvement in the renal capsules (Fig. 4).

### Histopathological scoring and average scores

The control group exhibited an average histopathological score of 0.2, indicating minimal changes when compared to the reference; the histopathological findings were characterized by minimal alterations, including slight brush border loss. Likewise, the EEE administered group displayed an average score of 0.2 which remained consistent with that of control group, suggesting the safe effect of EEE as no significant histopathological changes were noticed. The bifenthrin intoxicated group showed a higher average score of 1.6, signifying marked histopathological changes compared to either the control or EEE groups; the indications in this group were severe, encompassing glomerular atrophy, pyknotic nuclei, proteinaceous casts in the renal tubules, and dilatation of Bowman's capsule. Similarly, the group ingested with EEE prior to bifenthrin exhibited an average score of 1.1; the findings here were moderate in nature, characterized by loss of brush border observed in various fields; favorably, tubular dilatation was observed across multiple fields, highlighting a noticeable improving effect compared to the bifenthrin intoxicated group (Table 5).

**Table 5:** Histopathological scoring and average scores for different experimental groups.

Group	Field 1	Field 2	Field 3	Field 4	Field 5	Field 6	Field 7	Field 8	Field 9	Field 10	Average score
Control	0	0	1	0	0	1	0	0	0	0	0.2
Extract	0	0	0	0	0	0	0	1	0	1	0.2
Bifenthrin	1	1	2	1	3	1	3	2	1	1	1.6
Treated	1	1	1	2	1	0	1	2	1	1	1.1

## Discussion

Insecticide toxicity has been extensively researched in experimental animals using metabolic and histo-architectural indicators of organ toxicity^23^. Exposure to bifenthrin has been shown to increase renal pro-inflammatory cytokines (TNF-α, IL-2, and IL-6), hepato-renal oxidative stress, LDL, LDL-apoB-100, oxidized-LDL, and circulating cholesterol. All these conditions of stress must be closely associated with glomerular and renal tubular damage^24,25^. The precise mechanism of bifenthrin and other pyrethroid pesticides induced renal toxicity is unknown; despite this, animal studies have connected pyrethroid exposure to elevated catecholamines levels lead to release of renin which stimulates the renin-angiotensin system that result in production of vasopressin, and vasoconstriction of the glomerular arterioles consequently^17^.

Oxidative stress is a sensitive indicator that is frequently utilized for toxicological evaluations of pyrethroids, particularly bifenthrin, and other insecticides as a potential mechanism underlying their harmful effects^2^; however, numerous pyrethroids have been found to cause lipid peroxidative damage in different tissues^26^. In the current study, the values of the antioxidant markers (GSH, GPx and SOD) markedly decreased coupled with noticeable elevation in the level of the oxidative products (MDA and NO) in the renal tissue of bifenthrin-intoxicated group. These findings are consistent with the previous study of Dar et al.^24^. It was reported that reactive oxygen species (ROS) are produced because of the metabolism of xenobiotics, which includes pyrethroids; the body’s antioxidant defense is overwhelmed by the excessive creation of ROS, causing oxidative stress and inflammatory signs that leads to cellular and subcellular abnormalities^27^. The progression of chronic kidney disease (CKD) was found closely associated with systemic inflammation and oxidative stress, which are responsible for the manifestation of numerous complications^28^.

The TNF-α and IL-1β are generated by macrophages and monocytes; however they are released into the bloodstream, where they exert a systemic impact. TNF-α constitutes one of the first cytokines to be released during an inflammatory response^4^ and promotes the synthesis of IL-1β that occurs during prolonged oxidative stress^29^. IL-1β is a signaling molecule that modulates immunological response, facilitates leukocyte and lymphocyte activity, functions as a pyrogen, and promotes the progress of prolonged inflammation^30^.

The present study performed significant increases in the level of the inflammatory (IL-1β, TNF-α, IFN-γ) and apoptotic (caspase-3) markers in bifenthrin-intoxicated group; this result is consistent with that of previous study^9^ that demonstrated that low doses of bifenthrin can lead to a significant increase in the level of inflammatory cytokines in the kidney. Similarly, Jin et al.^31^ demonstrated the immunotoxic effects of bifenthrin on Zebrafish embryos which performed higher IL8, IL-1β, and Caspase 3 &9 levels.

Wang et al.^32,33^ studied the immunotoxic impact of bifenthrin on male mice. They gave light on the mechanisms of the immunotoxicity of bifenthrin through enhanced transcription concentrations of interleukin 6 and TNFα due to lipopolysaccharide stimulation, elevated ROS, and resultant oxidative stress-related gene dysregulation.

The observed elevated serum urea and creatinine levels post bifenthrin intoxication, indicating renal impairments. This observation runs in line with recent studies^8,34^. Nephrotoxicity endpoints modification may be supported by the idea that oxidative stress generated by ROS affects kidney macromolecules such as lipids, DNA, and protein, producing inflammatory, functional, and structural impairments^34^.

Moreover, the present study declared that bifenthrin intoxication resulted increased DNA-fragmentation ratio; this finding runs in line with Osman et al.^35^ who illustrated that bifenthrin caused different types of aberrations in Egyptian toads.

Research into the antitoxic ability of diverse medicinal plant extracts is a somewhat recent field of study; however, the benefits of utilizing phytomedicinal remedies vary between their low cost, free availability, and lack of possible health concerns associated with synthetic medicines^36^.

HPLC analysis of EEE revealed identification of 19 compounds that are characterized as having an antioxidative capacity including chlorogenic acid, naringenin, gallic acid coumaric acid, caffeic acid, querectin, rutin, and apigenin which were the major ingredients present in the extract. For examples, chlorogenic acid was found to improve hyperuricemia, alleviate renal inflammation, and ameliorate intestinal homeostasis in hyperuricemic mice^37^.

Khan et al.^38^ reported the protective effect of naringenin against doxorubicin-induced renal injury by down-regulating the levels of nuclear factor-κB (NF-κB), TNF-α and prostaglandin-E2. Gholamine et al.^39^ demonstrated the ameliorative effect of the gallic acid against oxidative stress mediating renal and hepatic injuries induced by sodium arsenite toxicity. It improved also hematological and histopathological changing induced by sodium arsenite. Investigators have described the renal anti-inflammatory and antioxidant activity of p**-**coumaric acid which has been found to attenuate oxidative stress and nephropathy in diabetic rats^40^ and ameliorate the biochemical and histopathological changes induced by gentamicin^41^. Gu et al.^42^ reported that quercetin can protect the kidney cells from COVID-19 injury by inhibiting inflammatory and apoptosis-related signaling pathways. Kandemir et al.^43^ reported the protective effect of rutin against sodium valproate-induce renal and hepatic damage and concluded that rutin exerts its action by inhibiting inflammation, apoptosis and autophagy. Apigenin was also found to protect kidney against doxorubicin-induced renal injury through inhibiting inflammation and oxidative stress^44^.

The current study demonstrated that EEE administration alone lowered oxidative stress markers and activated the antioxidant state in the rat kidney. In addition, co-administration of EEE one-hour pre-bifenthrin intoxication caused a significant elevation of the renal levels of the antioxidant battery (GSH, SOD and GPx), matched with a remarkable down-regulation in oxidative voltage (MDA and NO); this result agrees with Karhib et al.^45^ who stated that elevated level of GSH in kidney tissue protects cellular proteins from oxidation via the glutathione redox cycle, in addition to removing ROS. Also, EEE affects antioxidant enzymes that work together to protect against ROS. They attributed this effect to *E. purpurea* chelating property, which allows it to react with free radicals or highly reactive lipid peroxidation byproducts, as well as the enhancement of tissue thiol pools. This effect could be related to the high content of flavonoids and phenolic compounds which were proven to have anti-inflammatory and antioxidant effects^27,46^**.**

In a promising manner, this study showed that administration of EEE in line with bifenthrin led to downregulation in the pro-inflammatory (IL-1β, TNF-α, IFN-γ) and apoptotic (caspase-3) markers in the renal tissue reflecting its anti-inflammatory potential; Yu et al.^47^ and Karhib et al.^45^ attributed their same findings to the antioxidant properties of* E. purpurea* phytochemical constituents.

In addition, serum ALAT and ASAT activities as well as urea and creatinine levels of the bifenthrin-treated animals were significantly higher, demonstrating hepatotoxicity resulting from the leakage of these enzymes from the degenerated hepatocyte cytosol into the circulation^48^. Transaminase activities can be increased as well because of aggressive catabolism of amino acids to meet the immediate requirement for energy during pyrethroid stress^49^. Because* E. purpurea* extract comprises antioxidant ingredients such as phenolic and flavonoids components that are capable of suppressing lipid peroxidation, stabilizing cell membranes, preventing membrane lipids oxidation, and restoring cellular integrity, an improvement in the was noticed in the activity of these transaminases as previously explained^15^. Similarly, the reduced level of DNA damage by EEE may be attributed to its antioxidant activity that was monitored from a recent in vitro study, as EEE performed higher reducing power ability and radical scavenging activity^3^.

The results of the histopathological, morphometric and histopathological scoring confirmed the biochemical findings reported earlier and indicated that bifenthrin induced severe histological changes in the kidney tissues. Similar histological changes in the kidney have been documented previously^50^**.** However, animals treated with EEE resulted in an improvement in renal tissues as compared with the bifenthrin-intoxicated group, as EEE demonstrated improvement in the pathological changes. Morphometric and histopathological scoring assessment also showed an improvement in the glomerular and Bowman’s space and Malpighia’s corpuscle areas, as well as average scoring compared to that of rats intoxicated with bifenthrin only. These findings suggest possible modifications in the function and structure of the kidney which occurred probably by preventing oxidative stress. Wang et al.^51^ discovered that EEE’s caffeic acid is an additional biologically active component capable of inhibiting oxidative stress through elevating GSH and CAT values as well as suppressing inflammation by inhibiting TNF-α, IL-6, MAPK, phosphorated p38, and NF-κB, that was able to hold apoptosis through controlling caspase-3 and p53 concentrations.

Previous studies also indicated other alkylamide compounds that may also play a role in the EEE activity. Alkylamides are regarded to bneurale the key ingredients responsible for *Echinacea* plants anti-inflammatory effects^52^. Several investigations have shown that alkylamide-rich Echinacea extracts can influence pro-inflammatory cytokines such as TNF-α^53^ and have been demonstrated to have immunomodulatory properties in both *in vivo* and in vitro. These immunomodulatory impacts are thought to be related to alkylamides capacity for binding to cannabinoid receptor type 2^54^.

Despite the alkylamide fraction does not have antioxidative activity by itself, it boosts the ability of cichoric acid, a caffeic acid derivatives, which has been demonstrated to be responsible for most of EEE antioxidant activity since it is an effective scavenger of free radicals^55^. This explanation agreed with Wang et al.^56^ and Mohamed et al.^57^ who demonstrated that EEE cichoric acid reduced oxidative stress by tilting the balance of CAT, Nrf2, and GSH over MDA, resulting in reductions of apoptosis, neuronal damage, and inflammation. Ultimately, the antioxidant and anti-inflammatory phytochemical constituents of EEE inhibits and stabilizes the bifenthrin-induced free radicals and/or reactive oxygen species, and downregulate the inflammatory pathways, consequently. This behavior substantially reduced renal dysfunction (marked drop in the level of serum urea and creatinine) and pathological alterations in bifenthrin-poisoned rats, maintaining the antioxidant barrier and modifying renal-related pathways. Finally, EEE supplementation dramatically reduced tissue injury resulted from bifenthrin poisoning and was shown to be useful in reducing oxidative stress due to its potent antioxidant and chelating activities.

## Conclusion

In conclusion, the current study findings demonstrated that *Echinacea purpurea* ethanolic extract (EEE) has promising ameliorative, antioxidant, anti-inflammatory, renoprotective, and detoxifying efficiencies against bifenthrin-induced renal injury, as it succeeded in restoration of bifenthrin induced renal-dysfunction and pathological deteriorations. This effect could be attributed to the antioxidant activity of the EEE constituents. The extract could be recommended as a protective supplementary for bifenthrin-exposures after preclinical studies.

## Data Availability

All data generated or analyzed during this study are included in this published article.
